# Deep Learning-Based Nuclei Segmentation and Melanoma Detection in Skin Histopathological Image Using Test Image Augmentation and Ensemble Model

**DOI:** 10.3390/jimaging11080274

**Published:** 2025-08-15

**Authors:** Mohammadesmaeil Akbarpour, Hamed Fazlollahiaghamalek, Mahdi Barati, Mehrdad Hashemi Kamangar, Mrinal Mandal

**Affiliations:** 1 Electrical and Computer Engineering, University of Alberta, Edmonton AB T6G 2R3, Canada; barati@ualberta.ca (M.B.); mmandal@ualberta.ca (M.M.); 2 Faculty of Engineering and Technology, Shomal University, Amol 4616184596, Iran; mh.kamangar@shomal.ac.ir; 3 Electrical and Computer Engineering, University of Birjand, Birjand 9717434765, Iran; hamed.fazlollahi@birjand.ac.ir

**Keywords:** histopathological images, deep neural networks, nuclei segmentation, melanoma detection

## Abstract

Histopathological images play a crucial role in diagnosing skin cancer. However, due to the very large size of digital histopathological images (typically in the order of billion pixels), manual image analysis is tedious and time-consuming. Therefore, there has been significant interest in developing Artificial Intelligence (AI)-enabled computer-aided diagnosis (CAD) techniques for skin cancer detection. Due to the diversity of uncertain cell boundaries, automated nuclei segmentation of histopathological images remains challenging. Automating the identification of abnormal cell nuclei and analyzing their distribution across multiple tissue sections can significantly expedite comprehensive diagnostic assessments. In this paper, a deep neural network (DNN)-based technique is proposed to segment nuclei and detect melanoma in histopathological images. To achieve a robust performance, a test image is first augmented by various geometric operations. The augmented images are then passed through the DNN and the individual outputs are combined to obtain the final nuclei-segmented image. A morphological technique is then applied on the nuclei-segmented image to detect the melanoma region in the image. Experimental results show that the proposed technique can achieve a Dice score of 91.61% and 87.9% for nuclei segmentation and melanoma detection, respectively.

## 1. Introduction

Cutaneous Malignant Melanoma is one of the most severe skin cancers globally, causing many deaths each year. Early detection of skin cancer can significantly boost the chances of treatment and increase the survival rate. Digital histopathological image analysis plays an important role in cancer diagnosis. As histopathological images are typically very large, and due to the similar appearance of melanoma and non-melanoma, the manual procedure is challenging and time-consuming. In recent years, computer-aided diagnosis (CAD) has gained much popularity to help doctors speed up the diagnosis process [1]. Cells are the basic units of a biological structure and nuclei are very important parts of cells. Cancer affects the size and morphology of nuclei and other indicators, which can be used for cancer detection [2,3]. In image analysis, nuclei segmentation and classification are helpful in cancer diagnosis.

To identify different cell structures, the histopathological images are generally stained. The Hematoxylin and Eosin (H&E) is a very popular stain in histopathology because they vividly highlight the morphological features of nuclei and cytoplasm. In these H&E-stained images, cell nuclei (which contain chromatin) usually appear in shades of blue, whereas the cytoplasm and various connective tissues are displayed in different shades of pink. H&E-stained histopathological images vary in terms of color and shapes. Therefore, feature extraction, which forms the foundation of traditional image processing methods, becomes particularly challenging in such cases. There are many traditional image processing models to segment nuclei, such as applying threshold [4], filtering [5], and line scanning [6]. By developing traditional machine learning models, different methods in clustering, such as K-means [7], fuzzy C-means [8], and Support Vector Machines (SVMs) [9], have been introduced for image segmentation. These methods are based on pixels or small patch classification. In patch classification, proper patch selection is very important to achieve good results [10]. Traditional methods are based on feature extraction, but due to the diversity of shapes and colors in histopathological images of nuclei, proper feature extraction is very difficult [11]. However, due to the complexity and variability of nuclei in histopathological images, traditional methods often fall short in terms of accuracy and robustness, highlighting the need for more advanced approaches.

Machine learning and deep learning networks have recently become very popular in medical image analysis [12]. The Convolutional Neural Network (CNN) has emerged as a very popular deep learning model [13]. One of the main applications of CNNs in medical application is image segmentation [14]. CNNs demonstrate excellent results, but their high computational cost is a significant problem. To solve this problem of CNNs, the U-Net network [15] was introduced. U-Net includes more high-resolution and classification features produced in convolutions as supplements to the upsampling directly, which greatly improves the resolution in the image restoration stage. Many recent research is based on U-Net improvement [16] and its vast application range, like image segmentation [17] and image enhancement [18]. To enhance the feature expression abilities of pathological images, researchers have proposed multiple methods, including the introduction of the residual module, a multi-scale feature extraction module, attention mechanism, and the multi-model combination approach.

The objective of this paper is to present a robust nuclei segmentation and melanoma detection technique based on skin histopathological images. The paper is organized as follows. Section 2 presents a literature review and paper contributions. Section 3 presents the materials and the proposed method. Section 4 presents the experimental results. Section 5 presents a discussion of the results and limitations of the proposed method, followed by conclusions in Section 6.

## 2. Background

In recent years, CNNs have become widely used in medical image processing due to their powerful ability to diagnose different diseases. In particular, CNN-based methods have been extensively applied for the segmentation and analysis of cancer cell nuclei and tumor regions, which are critical for accurate diagnosis and treatment planning. They have been widely used not only for disease diagnosis via image segmentation, but also for identifying various regions and multiple types of abnormalities in medical images [19]. Table 1 presents various CNN-based methods that have been applied to image segmentation. Among these, particular attention is given to approaches specifically developed for nuclei segmentation. Sun et al. [20] proposed an automated convolutional framework (hereinafter referred to as ACF-Net) to segment nuclei in histopathological images. In this method, the main block is a “Deep Attention Integrated Network (DIANet)”, which uses VGG-16 as a feature extractor; and self-attention-based channel and spatial attention modules are also integrated. This block is introduced to obtain the relationships among the different feature regions, thereby achieving global perception and enhancing the relationships of different feature parts. In this paper, ACF-net is applied on datasets to segment and classify nuclei in H&E-stained histopathological images.

Shi et al. [21] proposed a CNN-based method called Automated Feature Global Delivery Connection Network (henceforth referred to as FGDC-Net) to segment nuclei in histopathological images. The main block in this method is the FGDC module, which involves convolutional layers, average pooling, and residual networks. This method enhanced the U-Net model by removing jumping connections and adding connections between adjacent layers to assign weights to intralayer feature channels of each layer to achieve better results. Zeng et al. [22] added the techniques of residual blocks as well as multi-scale and channel attention mechanisms to a U-net-based neural network for nuclei segmentation. Pan et al. [23] proposed an extension of U-Net with atrous depth-wise separable convolution for nuclei segmentation.

Alheejawi et al. [24] proposed an Improved Nuclei Segmentation network (henceforth referred to as INS-Net) to segment and classify cell nuclei in histopathological images. The segmentation architecture includes three parallel branches: a skip connection, Path A (which includes five convolutional layers) to extract coarse features; Path B (which includes twelve convolutional layers) to extract fine features; and Path C, which uses a skip connection. The outputs of the three paths are concatenated and the final segmented image is generated.

Different methods are introduced to segment nuclei based on a U-Net model. Li et al. [25] added a cascade residual fusion block to enhance the detection performance during the decoding process. Wan et al. [26] added a modified atrous spatial pyramid pooling to a U-Net model to capture multi-scale nuclei features and obtain nuclei context information without reducing the spatial resolution. Saha et al. [27] modified convolution layers, max-pooling layers, and deconvolution layers of a U-Net model; they also added spatial pyramid pooling layers and trapezoidal long short-term memory to the U-Net model.

Shorfuzzaman [28] proposed an explainable CNN-based stacked ensemble framework to detect melanoma skin cancer at earlier stages. This framework uses transfer learning where multiple CNN sub-models performing the same classification task are assembled. The model uses all sub-models’ predictions to generate the final prediction result. Djenouri et al. [29] used different deep learning architectures (VGG16, RESNET, and DenseNet) with ensemble learning and attention mechanisms to study interactions between different biomedical data for disease detection and diagnosis. In the ensemble model, different models are applied to one input image, and the final decision is made by evaluating the outputs. In the ensemble method, different CNN-based models are applied [30,31], but in the proposed model, one CNN-based model is applied to different images extracted from one input image to produce different outputs.

### Benefits of the Study

In this paper, an enhanced INS-Net model combined with a proposed ensemble strategy (henceforth referred to as ECE-Net) is proposed to segment nuclei and detect melanoma in skin histopathological images. In the proposed model, first, data augmentation in the test stage is applied to produce different images from one input image. Then, an enhanced INS-Net model is introduced to apply to the augmented images. In fact, in the proposed ensemble strategy, instead of combining predictions from multiple different models, multiple augmented versions of the input image are processed using the same model, and their outputs are aggregated to obtain the final result. Finally, averaging and voting ensemble techniques are used for the final classification of each pixel. The three main contributions of the paper are as follows:

- A novel data augmentation technique in the testing stage.
- An enhanced INS-Net as an improved Convolutional Neural Network model.
- An efficient ensemble technique for calculating final results.

## 3. Materials and Methods

**Figure 1:**
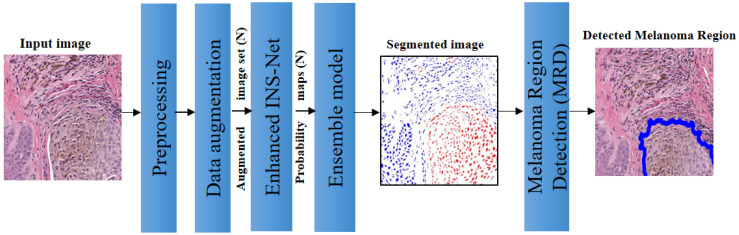
Schematic of the proposed ECE-Net model.

In this section, we present the materials and method proposed in this paper. Section 3.1 presents the dataset considered in this paper. Section 3.2, Section 3.3, Section 3.4, Section 3.5 and Section 3.6 present the proposed ECE-Net. Section 3.7 presents the performance evaluation metrics. The details of the proposed model is presented below. Figure 1 shows the schematic of the proposed model. As illustrated in Figure 1, the proposed model involves five modules: preprocessing, data augmentation, enhanced INS-Net, ensemble model (averaging or voting), and melanoma region detection (MRD).

As shown in Figure 1, the process begins with preprocessing to address color inconsistencies through color normalization. This is followed by data augmentation, which generates four rotated images and one enhanced image using a Gaussian filter, resulting in a total of five images. Subsequently, CNN and ensemble models are applied for nuclei segmentation. For melanoma region detection, a morphological processing step is employed as the final module.

### 3.1. Dataset

The digitized biopsies used in this study were obtained from the Cross Cancer Institute, University of Alberta, Edmonton, Canada, following the protocol for examining specimens with skin melanoma. The dataset included 15 large images and 100 histopathological images. The large images are approximately 5000×5000 pixels in size. There are 100 medium-sized images, each with a resolution of 960×960 pixels. These 100 images are partitioned into training (70 images), testing (15 images), and validation (15 images) datasets. To alleviate the computational cost associated with utilizing the entire image as input for the CNN, each image is segmented into non-overlapping blocks of 64×64 color pixels.

### 3.2. Preprocessing

Color normalization is used in the preprocessing stage. Normalization aids in speeding up the convergence of optimization algorithms during training, leading to faster and more stable learning outcomes.

Overall, the normalization of image datasets is a crucial preprocessing step that enhances the performance and generalization ability of machine learning models. The normalized pixel value at coordinate (m,n) in the color channel *c* is calculated as Equation (1):

(1) $$X_{\mathrm{norm}}\left(m,n,c\right)=\frac{X\left(m,n,c\right)-\mu_{c}}{\delta_{c}}$$

where X(m,n,c) is the gray value of the pixel, $\mu_{c}$ is the global mean of channel *c*, and $\delta_{c}$ is the global standard deviation of channel *c*.

### 3.3. Data Augmentation (DA)

**Figure 2:**
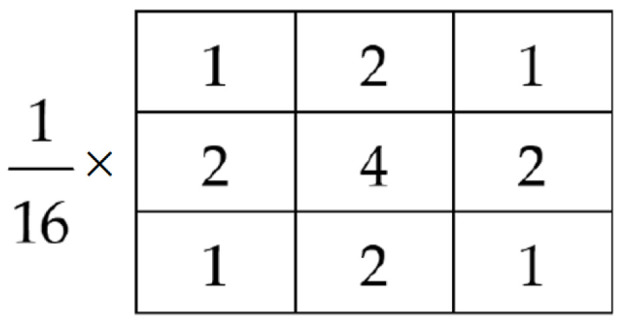
A 3 × 3 Gaussian mean filter *h* used in the proposed method for image enhancement.

**Figure 3:**
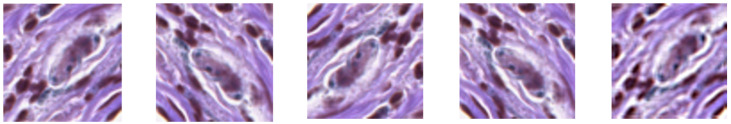
Example of an augmented image set. Left to right: Original test input image, 90-degree-, 180-degree- and 270-degree-clockwise-rotated images, edge-enhanced image.

Given an input image, the data augmentation (DA) module generates four additional images. Three images are generated by rotating the input image clockwise by $90^{\circ }$, $180^{\circ }$, and $270^{\circ }$. Let these images be denoted by $X_{90}$, $X_{180}$, and $X_{270}$. The fourth augmented image is an enhanced image, which is generated as Equation (2):

(2) $$X_{\mathrm{enh}}=X*h+\alpha X_{\mathrm{edge}}$$

where *X* is the input color image, *h* is a 3×3 Gaussian filter (shown in Figure 2), and $X_{\mathrm{Edge}}$ represents the edges of image *X*. Note that * is the convolution operator, and the filtering (performed separately on each color channel) is used to reduce the noise in the image. $X_{\mathrm{Edge}}$ is a binary edge image (edge: 1, non-edge: 0) obtained using the Canny edge detector. In our simulation, we have used α=20. Note that data augmentation is typically used when the training dataset is small. In this work, the data augmentation is applied on testing images to make the inference more robust. Figure 3 shows an augmented image set.

### 3.4. Enhanced INS-Net

**Figure 4:**
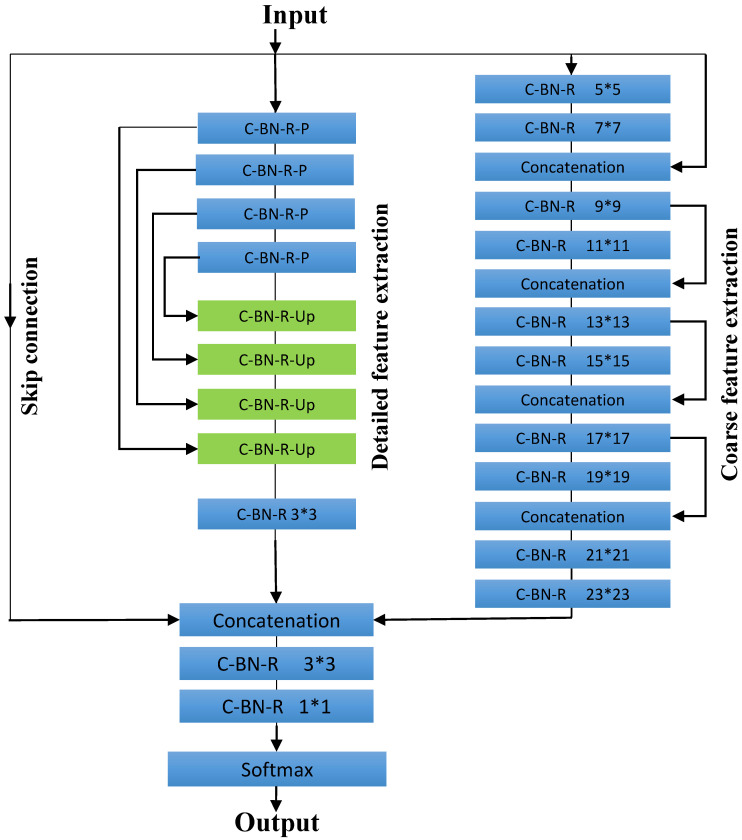
Architecture of the proposed ECE-Net model.

The schematic of the proposed DNN (enhanced INS-Net) is shown in Figure 4. The DNN consists of two paths: Detailed Feature Extraction (DFE) path and Coarse Feature Extraction (CFE) path. Out of 21 layers, 9 layers are in the DFE path and 10 layers are in the CFE path. Table 2 explains the different layer types of the proposed DNN. There are five types of layers in the proposed DNN, and the details of these layers are explained in Table 2.

As shown in Table 2, the proposed DNN model has five important layers: C-BN-R, C-BN-R-P, C-BN-R-UnP, Concatenate, and Softmax. In the Detailed Feature Extraction (DFE) path, after every two C-BN-R layers, there is one concatenation layer. This path is employed to extract detailed features from input images to depict melanoma, non-melanoma, and background regions (Related up-sampling layers are shown in green color in this path). It is important to note that in this path, no pooling layer is utilized; also, the output image is the same size as the input images. The Coarse Feature Extraction (CFE) path utilizes a U-Net-shaped model. This block incorporates four downsampling layers and four upsampling layers. To enhance the quality of results, the output of this path is integrated with the Detailed Feature Extraction block in the fusion block.

At the end of these paths, the two feature extraction paths, along with the skip connections, are concatenated in the concatenation module. In the prediction stage, the Softmax function serves as the final activation function of the neural network. It normalizes the network’s output into a probability distribution across the predicted output classes, where $P_{i}\left(m,n,c\right)$ denotes the probability of pixel (m,n) belonging to class *c*.

Note that there are three output classes: melanoma nuclei, non-melanoma nuclei, and background (i.e., non-nuclei pixels). Further, note that, as there is a set of five augmented images for each input image, there will be five probability matrices $P_{i}\left(m,n,c\right)$, one for each augmented image. Let these matrices be denoted by $P_{i}\left(m,n,c\right)$, where i=1,2,3,4,5.

Note that the DNN is an extension of the INS-Net [24] model with an improvement. The Detailed Feature Extraction (DFE) path is a modified version of Path B from the INS-Net architecture. However, unlike INS-Net, after every two C-BN-R layers, there is one concatenation layer. In this path, the number of C-BN-R layers is reduced, which significantly increases the model’s speed. To compensate for the reduction in C-BN-R layers, the number of concatenation layers is increased. An extra skip connection layer is introduced to assist the model in extracting more detailed feature maps. By increasing the number of concatenation layers, information from various parts of the network is combined, allowing the model to capture diverse features and patterns. The Coarse Feature Extraction (CFE) path utilizes a U-Net-shaped model. Unlike the INS-Net architecture, this block incorporates four downsampling layers and four upsampling layers. Table 3 compares the layers between the DNN of the INS-Net architecture and the proposed model.

### 3.5. Ensemble Model

For an M×N input image, the overall output of the DNN consists of five probability matrices: $\left\{P_{i}\left(m,n,c\right),\;i=1,2,3,4,5\right\}$. Note that *c* corresponds to the pixel classes: melanoma (c=1), non-melanoma (c=2), and background (c=3). In the proposed ensemble model, averaging and voting techniques [29] predict the output class of a pixel (m,n) of an input image based on these five matrices. The steps of the voting algorithm are as follows:

- Determine the class of the *i*th augmented image: $V_{i}\left(m,n\right)=\arg\max_{c}\left\{P_{i}\left(m,n,c\right)\right\},\;1\leq i\leq 5$.
- For each pixel (m,n), five classes are obtained from the augmented images. If there is a tie, the average of 5 neighboring pixels is considered as the pixel class. For the pixel (m,n), the pixel class is determined based on a majority vote. If there is a tie, the averaging algorithm is used to break the tie and determine the class. Let the overall class matrix be denoted by $V_{c}\left(m,n\right)$.

Note that the $P_{i}\left(m,n,c\right)$ and $P_{a}v\left(m,n,c\right)$ are probability matrices shown as continuous-tone color images, where the amount of blue, red, and green colors in the image for each pixel is proportional to the probability of that class (red, green, and blue channels correspond to classes 1, 2, and 3, respectively). The $V_{i}\left(m,n\right)$ are classified matrices shown as color images, where the red, green, and blue channels correspond to classes 1, 2, and 3, respectively. The steps of the averaging algorithm are as follows:

- Calculate the average probability matrix ($P_{\mathrm{av}}$) as Equation (3):
  (3) $$P_{\mathrm{av}}\left(m,n,c\right)=\frac{1}{5}\sum_{i=1}^{5}P_{i}\left(m,n,c\right)$$
- For each pixel (m,n), calculate the class matrix ($M_{c}$) by choosing the class for which $P_{\mathrm{av}}\left(m,n,c\right)$ is maximum as Equation (4):
  (4) $$M_{c}\left(m,n\right)=\arg\max_{c}\left(P_{\mathrm{av}}\left(m,n,c\right)\right)$$

Note that the $P_{i}\left(m,n,c\right)$ and $P_{\mathrm{av}}\left(m,n,c\right)$ are probability matrices shown as continuous-tone color images, where the amount of blue, red, and green colors in the image for each pixel is proportional to the probability of that class (red, green, and blue channels correspond to classes 1, 2, and 3, respectively). Also, $M_{c}\left(m,n\right)$ is a classified matrix shown as a color image, where the red, green, and blue channels correspond to classes 1, 2, and 3, respectively.

### 3.6. Melanoma Region Detection (MRD)

This block uses melanoma masks to extract melanoma regions from the original image. The process involves several morphological operations: dilation, image filling, erosion, and threshold.

### 3.7. Evaluation Metrics

The performance measures used in this paper are accuracy, precision, recall, Dice coefficient, and Jaccard Score, as shown in Equations (5)–(9):

(5) $$\mathrm{Accuracy}=\frac{TP+TN}{TP+TN+FP+FN}$$

(6) $$\mathrm{Precision}=\frac{TP}{TP+FP}$$

(7) $$\mathrm{Recall}=\frac{TP}{TP+FN}$$

(8) $$\mathrm{Dice}=\frac{2\times \mathrm{precision}\times \mathrm{recall}}{\mathrm{precision}+\mathrm{recall}}$$

(9) $$\mathrm{Jaccard}\;\mathrm{Score}=\frac{TP}{TP+FP+FN}$$

where *TP*, *TN*, *FN*, and *FP* refer to True Positive, True Negative, False Negative, and False Positive, respectively.

## 4. Results

In this section, the segmentation performance of the proposed method is compared with other methods. In this paper, we have implemented U-Net [15], FGDC-Net [21], INS-Net [24], and ACF-Net [20] as the state-of-the-art techniques. These techniques have been implemented in Python 3.11. They use different convolutional layers, skip layers, concatenation layers, and various blocks, all of which are implemented and trained using our images of size 64×64. The number of training images is the same for all methods. Our proposed method is also implemented in Python 3.11. In the proposed method, 22 convolutional layers and 7 skip connections are implemented. There are two main paths and one skip connection in our method. After concatenating the outputs of these paths and the skip connection, two convolutional layers are used to extract features. Finally, a softmax function produces the final output.

Both objective and subjective comparisons are performed in this section. After augmenting each segmented image (with size 64×64), the position of each pixel in all rotated and enhanced images is known. The class of each pixel from the original image and the corresponding pixel in all other four images (three rotated images and one enhanced image) is predicted. For each pixel, there are five predicted classes; averaging and voting methods are applied to identify the most frequently occurring class. The performance measures used in this paper are accuracy, precision, recall, Dice coefficient, and Jaccard Score.

### 4.1. Architecture Comparison

Table 4 shows configuration details of different recent methods used in performance evaluation. As shown in Table 4, the proposed model is simpler than other recent models.

### 4.2. Nuclei Segmentation Performance

**Figure 5:**
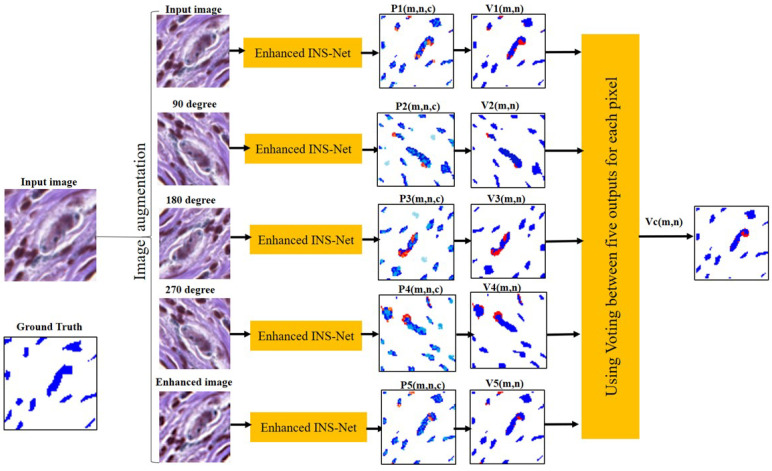
Schematic of the voting ensemble model for five images from Figure 3.

**Figure 6:**
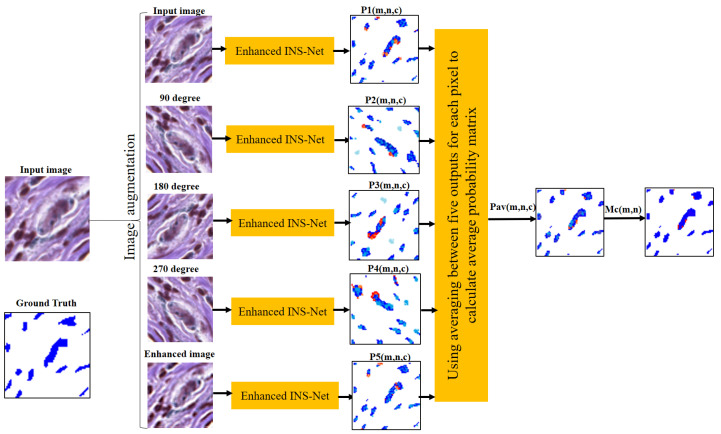
Schematic of the averaging ensemble model for five images from Figure 3.

As mentioned earlier, the final nuclei segmentation is performed by combining the individual segmentation masks (generated by each of the five augmented images) based on the voting or averaging method. The schematic of the voting technique used in the proposed method is shown in Figure 5. Details of the averaging technique used in the proposed method for one test input image are shown in Figure 6.

As shown in Figure 5 and Figure 6, the enhanced INS-Net is applied to perform segmentation on all produced images (three rotated images, one enhanced, and one original image). To enhance the accuracy of the segmentation for the original image, voting or averaging techniques are utilized in the ensemble model. Please note that all augmentations are used solely for predictions on the test data, this means that we initially train a model on the prepared dataset. For each pixel, there are different coordinates in each produced image. In the proposed method, all these new coordinates are saved as the corresponding pixel. For segmentation, each pixel classification result in the rotated images is compared with its corresponding pixels in the original image. As a final decision in classification, two techniques (averaging and voting) are applied to determine the final class of each pixel.

Since the primary class of interest is nuclei and all other pixels are categorized as the background, the model’s performance in accurately segmenting nuclei regions is crucial. Table 5 shows the confusion matrix for the ECE-Net model for an image with 12,590,651 pixels.

**Figure 7:**
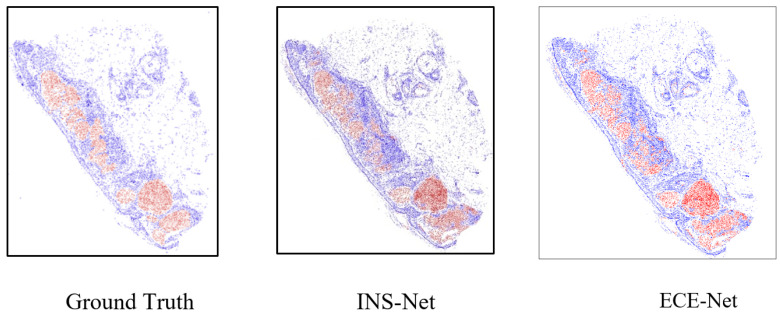
Visual comparison of melanoma detection between INS-Net and ECE-Net. Melanoma: Red, Non-Melanoma: Blue, Background: White.

For N=3, we consider the original image and its clockwise rotations by $90^{\circ }$ and $270^{\circ }$. As shown in Table 6, the proposed ECE-Net model performs better in compression compared to other recent models. Combining the proposed model with ensemble techniques (voting and averaging) during testing yields better results than using the original ECE-Net alone. Table 6 shows that the averaging ensemble outperforms the voting ensemble for both numbers of augmented images. Furthermore, the number of augmented images has a greater impact on performance than the choice of ensemble technique. Specifically, while the averaging technique performs better than voting with fewer augmented images (N=3), increasing the number of augmented images to five results in the voting technique outperforming the averaging technique with N=3. This demonstrates that the number of augmented images is more critical than the ensemble method used. As a visual comparison, the results are shown in Figure 7, where the proposed model is compared with INS-Net.

## 5. Discussion

**Figure 8:**
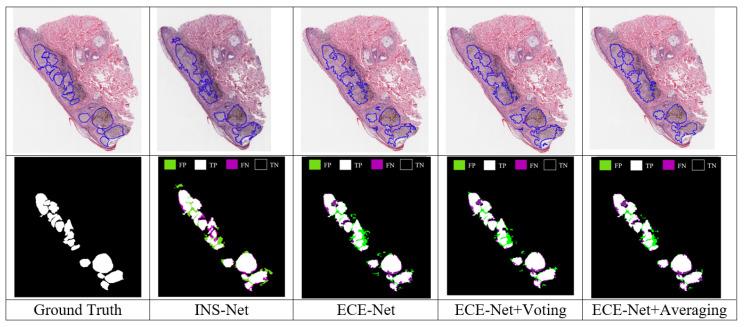
Visual comparison of the MRD module: INS-Net, ECE-Net, ECE-Net+voting, and ECE-Net+averaging, along with the ground truth.

To summarize, in this section, the comparison results between our proposed methods and other methods have been reported. The report and comparison are for final outputs after using the MRD module. The MRD module is visually evaluated in Figure 8. As shown in Figure 8, the proposed method has better performance in comparison to INS-Net. Also, as shown in Figure 8, using two voting and mean ensemble models produce better results in comparison to the proposed method. For a better understanding, related TPs, TNs, FPs and FNs are shown visually with white, black, green and purple colors, respectively.

The MRD module (consisting of dilation, image fill, erosion, and threshold operations) is evaluated based on various evaluation metrics, including accuracy, precision, recall, and Dice coefficient, as presented in Table 7. Notably, the order of results mirrors that of the nuclei segmentation results. Specifically, ECE-Net outperforms INS-Net but lags behind the ECE-Net + voting ensemble. The most favorable outcomes are observed for the ECE-Net + averaging ensemble. This superiority can be attributed to the fact that MRD utilizes masks generated by the nuclei-segmented images. Consequently, improved nuclei segmentation results yield superior melanoma region detection outcomes.

### Implications, Limitations, and Future Perspectives

This research is highly beneficial for computer-aided diagnosis, as it ultimately identifies cancerous regions, which can greatly assist medical specialists in disease detection. Since the study is based on real patient data from an Alberta hospital, it holds practical potential for real-world application. The main limitation is the computationally intensive nature of the processing, which currently prevents real-time diagnosis. However, by exploring and implementing newer CNN models and GPU accelerators, the system’s accuracy and processing speed can be further improved.

## 6. Conclusions

In this paper, a novel CNN-based model has been proposed for automatic segmentation of H&E-stained skin histopathological images into three classes: melanoma, non-melanoma, and background. The proposed method combines an enhanced INS-Net architecture with a special ensemble model to improve segmentation accuracy. Unlike traditional ensemble methods that combine outputs from different CNN architectures on one image, our proposed ensemble model applies a single CNN model to different augmented versions of the input image. This strategy significantly improves model robustness and accuracy. Experimental results demonstrate that the proposed method achieves a Dice coefficient of 91.61%, and Jaccard Score of 78.4% for nuclei segmentation outperforming state-of-the-art nuclei segmentation methods. For the melanoma region detection, the ECE Net achieves a Dice score of 87.9% and a Jaccard Score of 78.41%.

For future work, several directions can be explored to further enhance performance and applicability. First, expanding the augmentation techniques beyond simple rotations to include scaling, cropping, and image synthesis could help the model generalize better to varied histopathological patterns. Second, the ensemble framework could be enriched by incorporating newer and deeper neural network architectures, potentially improving prediction reliability and accuracy. These enhancements will not only improve the technical performance but also help bridge the gap between AI models and practical clinical deployment in melanoma diagnosis.

## Figures and Tables

**Table 1:** List of a few state-of-the-art nuclei segmentation and cancer detection techniques based on CNNs.

Ref.	Year	Application	Method	Dataset
[20]	2023	Nuclei segmentation	DAI-Net	Multi-organ
[21]	2022	Nuclei segmentation	FGDC-Net	Multi-organ
[22]	2019	Nuclei segmentation	RIC-UNET	Multi-organ TCGA
[23]	2019	Nuclei segmentation	AS-UNet	MOD and BNS
[24]	2021	Melanoma detection	INS-Net	Skin Dataset
[25]	2019	Colon cancer detection	Modify UNET	Colorectal
[26]	2020	Breast cancer detection	ASPPU-Net	breast histopathology
[27]	2018	Breast cancer detection	Modify UNET	Multi-organ dataset

**Table 2:** Different layer types in the proposed ECE-Net model shown in Figure 4.

Layers	Includes
C-BN-R	Conv2d layer, Batch Normalization, ReLU
C-BN-R-P	Conv2d layer, Batch Normalization, ReLU, pooling (2 × 2)
C-BN-R-UnP	Conv2d layer, Batch Normalization, upsample (2 × 2)
Concatenate	Combine the feature maps
SoftMax	Find the probability of classes for each pixel

**Table 3:** Comparison of the layers and parameters between the proposed ECE-Net model and INS-Net.

Path	CFE Path		DFE Path	
**Model**	**INS-Net**	**ECE-Net**	**INS-Net**	**ECE-Net**
No. of Convolutional layers	12	10	11	9
No. of Skip connections	3	4	0	0
No. of Training parameters	131,242	101,268	94,745	41,133
Filter size	(3×3)−(25×25)	(5×5)−(23×23)	(1×1)−(7×7)	(1×1)−(5×5)

**Table 4:** Configuration details of different CNN models used for melanoma detection.

CNN Architecture	Convolutional Layers	No. of Parameters	Filter Size	No. of Filter per Layer
U-Net [15]	11	905,472	3×3	(64, 128, 256)
ACF-Net [20]	31	228,096	1×1–3×3	(32, 64, 128, 256, 512)
FGDC-Net [21]	28	256,304	3×3–15×15	(64, 128, 256)
INS-Net [24]	18	225,987	3×3–21×21	64
ECE-Net (Proposed)	15	152,401	1×1–23×23	64

**Table 5:** Confusion matrix for the ECE-Net system for one large image with 12,590,651 pixels.

Actual Class	Predicted Nuclei (Number of Pixels)	Predicted Background (Number of Pixels)
nuclei	3,000,042	303,085
background	345,378	8,942,146

**Table 6:** Nuclei segmentation performance comparison between the proposed ECE-Net model and other related recent works.

Technique	Accuracy (%)	Precision (%)	Recall (%)	Dice Coefficient (%)
U-Net [15]	78.79	87.41	57.87	69.63
FGDC-Net [21]	93.63	88.97	87.30	88.13
INS-Net [24]	94.11	89.88	88.18	89.02
ACF-Net [20]	94.26	91.36	87.59	89.43
ECE-Net (without ensemble)	94.84	89.66	90.82	90.23
ECE-Net with voting ensemble (*N* = 3)	95.40	90.86	91.76	91.30
ECE-Net with average ensemble (*N* = 3)	95.47	91.01	91.85	91.43
ECE-Net with voting ensemble (*N* = 5)	95.51	91.10	91.92	91.51
ECE-Net with average ensemble (*N* = 5)	95.56	91.25	91.99	91.61

**Table 7:** Nuclei segmentation performance comparison.

Technique	Accuracy (%)	Precision (%)	Recall (%)	Dice Coefficient (%)	Jaccard Score (%)
INS-Net model [24]	97.7	83.22	87.08	85.1	74.07
ECE-Net model	97.82	85.96	87.2	86.58	76.33
ECE-Net + voting ensemble	97.96	86.82	88.08	87.45	77.69
ECE-Net + Ave. ensemble	98.03	87.39	88.41	87.9	78.41

## Data Availability

The data presented in this study are available on request from the corresponding author.

## Abbreviations

The following abbreviations are used in this manuscript:

AI	Artificial Intelligence
CAD	Computer-Aided Diagnostics
DNN	Deep Neural Network
H&E	Hematoxylin and Eosin
ACF-Net	Automated Convolutional Framework Network
DIANet	Deep Attention Integrated Network
FGDC-Net	Feature Global Delivery Connection Network
INS-Net	Improved Nuclei Segmentation Network
ECE-Net	Enhanced INS-Net Combined with Ensemble Model
VGG16	Visual Geometry Group 16-layer network
RESNET	Residual Network
DenseNet	Densely Connected Convolutional Network
MRD	Melanoma Region Detection
DFE	Detailed Feature Extraction
CFE	Coarse Feature Extraction
